# Advances in the clinical application of stellate ganglion block for non-pain indications

**DOI:** 10.3389/fmed.2026.1840174

**Published:** 2026-08-19

**Authors:** Yachao Wang, Hua Zhuo, Lingang Wang

**Affiliations:** Department of Anesthesiology, The First People’s Hospital of Xiaoshan District, Hangzhou, China

**Keywords:** hot flashes, long COVID, non-pain indications, Post-Traumatic Stress Disorder, stellate ganglion block, sympathetic blockade, ultrasound guidance, ventricular arrhythmias

## Abstract

**Background:**

Stellate ganglion block (SGB) is a peripheral nerve block technique involving injection of local anesthetics and/or steroids near the stellate ganglion. Traditionally used for pain-related syndromes, SGB has recently expanded into non-pain fields with the advancement of ultrasound guidance, showing therapeutic potential in arrhythmias, menopausal hot flashes, psychiatric disorders, cerebrovascular diseases, insomnia, and COVID-19 sequelae.

**Objective:**

To review the current evidence on SGB applications in non-pain conditions, evaluate its therapeutic efficacy and safety across different diseases, elucidate underlying mechanisms, and identify research gaps and future directions.

**Methods:**

This narrative review synthesized data from published literature including case reports, cohort studies, and randomized controlled trials (RCTs) on SGB in non-pain conditions. Studies were analyzed based on disease categories, with focus on mechanism of action, clinical outcomes, and safety profiles.

**Results:**

Stellate ganglion block demonstrates therapeutic effects across six domains: (1) Ventricular arrhythmias: In a multicenter observational study of 131 patients (the STAR study), 92% (106/115) of patients with treatable arrhythmic episodes achieved a ≥50% reduction in arrhythmia burden within 12 h via sympathetic blockade and QT dispersion reduction; (2) Menopausal hot flashes: Significant reduction in frequency through modulation of thermoregulatory pathways; (3) Insomnia: Improved sleep quality via autonomic balance restoration; (4) Cerebrovascular diseases: Enhanced cerebral perfusion and cognitive function; (5) Post-Traumatic Stress Disorder (PTSD): Case series have reported response rates of 70%–75% via inhibition of locus coeruleus-norepinephrine circuits; randomized evidence is mixed–a small early trial found no benefit over sham, whereas the subsequent multicenter randomized controlled trial (*n* = 113) demonstrated a statistically significant reduction in PTSD symptom severity (*P* = 0.01)–and adequately powered confirmatory trials are still needed; (6) COVID-19: Retrospective cohort studies suggest symptom relief in the majority of patients through autonomic reset.

**Conclusion:**

Stellate ganglion block has become an important intervention for autonomic dysfunction in non-pain conditions through sympathetic blockade, neuroendocrine modulation, and anti-inflammatory effects. Although ultrasound guidance has improved safety, evidence remains limited by small RCTs, heterogeneous protocols, and observational designs. Rigorous multicenter RCTs are needed to confirm efficacy, define patient selection, and standardize protocols. Further research should investigate central regulatory mechanisms and long-term neuroplasticity to advance SGB from empirical therapy to precision intervention.

## Introduction

The stellate ganglion is a sympathetic ganglion formed by the fusion of the inferior cervical ganglion and the first thoracic ganglion. Anatomically situated between the levels of the sixth (C6) and seventh (C7) cervical vertebrae, it provides postganglionic sympathetic innervation to the head and neck and contributes sympathetic fibers to the upper limbs and heart. Stellate ganglion block (SGB) is a specialized peripheral nerve block technique performed by injecting local anesthetics and/or corticosteroids into the vicinity of the stellate ganglion (1). This blockade technique has traditionally been utilized for the management of various pain-related syndromes. Given the pivotal role of the sympathetic nervous system in the pathophysiology of multiple chronic pain states, the suppression of sympathetic hyperactivity can enhance therapeutic efficacy (2). With recent advancements in ultrasound-guided techniques, the clinical application of SGB has expanded extensively into non-pain indications. It has demonstrated significant therapeutic efficacy in the management of cardiac arrhythmias, perimenopausal hot flashes, insomnia, cerebrovascular diseases, psychiatric disorders, and the sequelae of COVID-19 (Table 1). Beyond these domains, SGB has also been applied to hyperhidrosis, Raynaud’s phenomenon, and acute upper-limb ischemia–including ischemia caused by accidental intra-arterial injection of medication intended for intravenous administration.

**TABLE 1:** Therapeutic effects and medication protocols of stellate ganglion block in non-pain treatment.

Diseases		Mechanism of action	Clinical outcomes	Medication protocol and dosage	Adverse reactions	Landmark studies
Arrhythmias		Peripheral nerve blockade - electrophysiological stabilization - anti-inflammatory protection - central remodeling.	92% of patients experienced at least a 50% reduction in arrhythmic events (ATP/shock) within 12 h post-treatment.	Single injection: drug and dose at operator’s discretion in the STAR study (most common bolus regimens: lidocaine alone 28.8%, lidocaine + ropivacaine 34.2%, lidocaine + bupivacaine 23.9%). Continuous infusion (Sanghai et al.): loading dose of 5–10 mL of 0.5% ropivacaine, followed by 6–10 mL/h of 0.2% ropivacaine.	Reversible Horner syndrome (incidence 84%–100%) and hoarseness (10%)	(25)
Perimenopausal hot flashes		Peripheral sympathetic nerve blockade → central NGF/NE pathway regulation → functional remodeling of thermoregulatory nuclei.	Hot flash frequency decreased by 52% (6 months).	5 mL of 0.5% bupivacaine; or 10 mL of 0.25% levobupivacaine; or 5 mL of 0.375% bupivacaine	Reversible Horner syndrome	(40)
Insomnia		Restoration of autonomic balance and inhibition of HPA axis hyperactivation.	PSQI scores decreased by 8–12 points, and both sleep efficiency and total sleep time improved.	3–7 mL of 0.25%–0.5% ropivacaine.	Hoarseness/dysphagia	(42, 45, 46)
Cerebrovascular diseases		Inhibition of sympathetic vasoconstriction → modulation of vasoactive factors → improvement of macro- and microcirculatory perfusion → neuroprotection.	Significant improvement in cognitive and swallowing function; significant reduction in peak velocity of the middle cerebral artery.	6–8 mL of 1% mepivacaine; or 10 mL of 2% lidocaine; or 10 mL of 0.5% bupivacaine.	Horner syndrome	(56, 57)
Psychiatric disorders	PTSD	Inhibition of the LC-NE-BLA fear memory circuit → reduction of central noradrenergic drive → specific blockade of fear memory consolidation.	CAPS score decreased by 6.24 points.	5–7 mL of 0.5% ropivacaine.	Transient hoarseness (9.3%), dysphagia, and Horner syndrome; the incidence of severe complications such as pneumothorax or hematoma is less than 1%.	(61, 66)
	Anxiety and depression	Restoration of autonomic balance, inhibition of HPA axis hyperactivation, modulation of central monoamine neurotransmitters, and anti-inflammatory/antioxidant effects.	Significant reduction in anxiety and depression scale scores.	Daily alternating bilateral injections of 0.2% ropivacaine hydrochloride (5 mL) across three 6-session courses.	Hoarseness, dysphagia, and Horner syndrome	(73, 74)
COVID-19		Modulation of the autonomic-immune-vascular axis.	Effects on fatigue, brain fog, and headaches are relatively well-defined, though clinical efficacy exhibits significant heterogeneity.	0.5% bupivacaine combined with betamethasone; or a mixture of 0.25% bupivacaine and dexamethasone.	Worsening of headache and fatigue	(76, 81)

## Methods

### Search strategy and selection criteria

To construct this narrative review, a comprehensive literature search was conducted in electronic databases, including PubMed and Web of Science, from their inception to January 2026. The search strategy combined Medical Subject Headings (MeSH) and text words related to “stellate ganglion block,” “ultrasound guidance,” and non-pain indications such as “ventricular arrhythmias,” “hot flashes,” “Post-Traumatic Stress Disorder,” “insomnia,” and “long COVID.”

Studies were included if they met the following criteria: (1) discussed the clinical application, efficacy, or mechanism of SGB for non-pain conditions; and (2) were published in English as full-text articles, including randomized controlled trials, cohort studies, case series, and case reports. Non-peer-reviewed preprints, abstracts without full texts, and studies lacking clear clinical data were excluded. While a formal standardized quality assessment tool was not applied due to the broad scope and narrative nature of this review, the methodological strengths and limitations of the pivotal studies are critically discussed within the text.

### SGB and cardiac arrhythmias

The sympathetic nervous system is a pivotal mechanism in the genesis of ventricular arrhythmias and constitutes an important therapeutic target (3). Current therapeutic modalities, such as beta-blockers, antiarrhythmic agents, mechanical circulatory support, and catheter ablation, often exhibit limited efficacy in controlling ventricular flutter and fibrillation. Recurrence persists in a subset of patients despite multimodal management, and therapeutic options are further constrained in the presence of hemodynamic instability. Emerging evidence suggests that SGB, by attenuating cardiac sympathetic tone, represents an effective adjuvant therapy for the management of ventricular arrhythmias and electrical storms (4–8). SGB exerts anti-arrhythmic effects through multidimensional mechanisms, fundamentally by precisely interrupting cardiac sympathetic innervation and modulating autonomic balance. Specifically, SGB utilizes local anesthetics to reversibly blockade the cervicothoracic sympathetic chain (segments C5–T3), which inhibits the release of norepinephrine (NE) from postganglionic fibers, thereby attenuating cardiomyocyte excitability and automaticity (9–11). At the electrophysiological level, this blockade significantly prolongs the ventricular effective refractory period (VERP) and elevates the ventricular fibrillation threshold. It reduces the dispersion of action potential duration, thereby eliminating early after depolarizations (EADs) and the pathological substrate for reentry (9, 10, 12, 13). Left-sided SGB (LSGB) is considered the preferred intervention given the predominant role of the left stellate ganglion in the sympathetic innervation of the left ventricle. Mechanistically, LSGB inhibits L-type calcium currents and late sodium currents while restoring potassium current balance; this modulation shortens the QT interval and decreases QT dispersion, thereby effectively suppressing malignant arrhythmias, including Torsades de Pointes (14–17). Furthermore, SGB interrupts the vicious cycle of the “sympathetic storm” by inhibiting excessive catecholaminergic drive, thereby temporarily attenuating the catecholamine surge and providing a therapeutic window for definitive interventions such as catheter ablation (15, 18–20). Notably, the clinical effects of SGB often persist beyond the half-life of the local anesthetics, suggesting that its long-term benefits are mediated by the inhibition of retrograde transport of nerve growth factor, the attenuation of central sympathetic outflow, and the induction of central neural remodeling (13, 18, 19). Moreover, SGB demonstrates concomitant anti-inflammatory and cardioprotective effects. By reducing the concentrations of oxidative stress markers, plasma NE, and angiotensin II, it improves left ventricular function and optimizes perfusion in the ischemic myocardium (13, 21–24). In the STAR study, a multicenter international observational longitudinal study that enrolled 133 patients across 19 centers (131 analyzed), left-sided percutaneous SGB (98.4% of 184 procedures; performed via an anterior landmark approach in 57.6% and a lateral ultrasound-guided approach in 42.4% of procedures) resulted in a ≥50% reduction in arrhythmic events [anti-tachycardia pacing (ATP) and/or shocks] within 12 h in 92% (106/115) of the patients who had arrhythmic episodes requiring treatment in the preceding 12 h (median reduction 100%, IQR −100% to −92.3%). A single major complication (0.5% of procedures)–local anesthetic systemic toxicity with respiratory depression that resolved after lipid emulsion therapy–was reported (25). While these results are promising, the absence of a randomized controlled design and the relatively small sample size preclude definitive conclusions, and the precise percentage should be interpreted with caution. A systematic review of 23 published reports encompassing 38 patients further corroborated these findings: SGB significantly reduced the mean daily ventricular arrhythmia burden (12.4 ± 8.8 vs. 1.04 ± 2.12 episodes/day, *P* < 0.001) and the number of external and ICD shocks (10.0 ± 9.1 vs. 0.05 ± 0.22 shocks/day, *P* < 0.01) (26). In acute resuscitation settings, the bedside ultrasound-guided technique allows for procedure completion within 2.4 min; the injection of 10 mL of 1% lidocaine beneath the carotid sheath at the C6 vertebral level, performed during continuous chest compressions, can successfully terminate refractory ventricular fibrillation (10). Notably, continuous infusion protocols offer significantly enhanced efficacy compared with single-injection techniques. A regimen consisting of a loading dose of 5–10 mL of 0.5% ropivacaine, followed by a maintenance infusion of 6–10 mL/h of 0.2% ropivacaine, has been reported to reduce arrhythmia burden by 94% with a median duration of 3 days, thereby obviating the need for repeated blockade (6). SGB also functions as a bridge to surgical Left Cardiac Sympathetic Denervation (LCSD); continuous infusion protocols using indwelling catheters have been reported to maintain rhythm control for extended periods, obviating the need for repeated injections (6). In acute resuscitation settings, SGB can effectively suppress adrenergic triggers that perpetuate refractory ventricular arrhythmias. Morena et al. reported a case of fulminant acute myocarditis complicated by ventricular fibrillation refractory to multiple direct current shocks and antiarrhythmic medications; ultrasound-guided single-shot SGB with 8 mL of 0.375% ropivacaine successfully attenuated the sympathetic hyperactivity driven by myocardial inflammation, thereby restoring defibrillation effectiveness and achieving return of spontaneous circulation (ROSC) (27). This case underscores that SGB can interrupt the vicious cycle of “sympathetic storm” even when the trigger is non-ischemic inflammatory injury, providing a critical therapeutic window for definitive interventions. Regarding the safety profile, ultrasound guidance has reduced the incidence of severe complications to less than 0.5%. The procedure can be safely performed even in patients receiving dual antiplatelet or anticoagulation therapy (25). The primary adverse effects include reversible Horner syndrome (incidence 84%–100%) and hoarseness (approximately 10%) (13) (Table 2).

**TABLE 2:** Key clinical study characteristics of stellate ganglion block for the management of ventricular arrhythmias.

References	Study design	Sample size	Blockade approach	Medication protocol	Primary outcome	Efficacy data
Savastano et al. (25)	STAR	131	Left-sided percutaneous SGB (98.4% of procedures); anterior landmark approach 57.6%, lateral ultrasound-guided approach 42.4%; single bolus 82.6%, bolus + continuous infusion 17.4%.	Not protocol-specified (operator’s choice; lidocaine alone 28.8%, lidocaine + ropivacaine 34.2%, lidocaine + bupivacaine 23.9% of boluses)	A ≥50% reduction in arrhythmic events [antitachycardia pacing (ATP) and/or shocks] within 12 h post-intervention.	An overall efficacy rate of 92% was achieved, with a median reduction of 100%.
Sanghai et al. (6)	Single-center retrospective cohort study	18	Single injection versus continuous infusion.	0.5% ropivacaine (loading dose) and 0.2% ropivacaine (maintenance infusion)	The number of implantable cardioverter-defibrillator therapies and sustained VAs/24 h were compared between the pre-SGB and post-SGB periods.	Median VA/24 h reduction was 94% in the continuous-infusion group versus 45% in the single-injection group (*P* = 0.006 between groups); 7/9 versus 5/9 patients remained free of recurrent VA.
López-Millán Infantes et al. (12)	Observational study	7	Initially, a unilateral left-sided approach was performed; in the absence of a therapeutic response, a bilateral intervention was subsequently implemented.	Not reported	To evaluate the efficacy and safety of ultrasound-guided stellate ganglion block for the management of refractory electrical storm.	Control of arrhythmias (*p* = 0.018)

Current research, however, remains subject to several limitations. First, the existing evidence base is predominantly derived from case reports and cohort studies; although the STAR study provides the most extensive prospective observational data to date, the absence of a randomized controlled design precludes the complete exclusion of placebo effects (25); second, therapeutic efficacy exhibits heterogeneity: in a multicenter registry of 117 patients with refractory ventricular arrhythmias, 63.2% achieved a ≥50% reduction in 24-h arrhythmia burden after SGB (28). Age was the only significant predictor of success (OR 0.96 per year), with response rates of only 10% in patients over 75 years (29), moreover, non-ischemic cardiomyopathy, a history of ventricular arrhythmias, and longer ventricular tachycardia (VT) cycle lengths are associated with a higher risk of recurrence (30); furthermore, the duration of action is limited (2–5 h), necessitating repeated injections or conversion to continuous infusion in 45.3% of patients (28), which in turn increases the risks of catheter displacement (7%) and left upper limb weakness (9%) (31). Future studies should define optimal patient selection criteria and explore long-acting neurolytic techniques, such as pulsed or continuous radiofrequency ablation, to extend the duration of therapeutic action. Furthermore, the development of minimally invasive approaches, such as transtracheal cardiac plexus block, is warranted to expand indications to include critically ill patients presenting with cardiogenic shock complicated by electrical storm. Additionally, further investigation is required to elucidate the mechanisms underlying the anti-inflammatory effects and neural remodeling modulation of SGB, thereby accounting for clinical efficacy that persists beyond the half-life of local anesthetics. Finally, the establishment of standardized ultrasound-guided protocols is essential to further minimize complications.

### SGB and hot flashes

The fundamental mechanism underlying SGB in the management of hot flashes involves the interruption of pathological connectivity between the sympathetic nervous system and the thermoregulatory centers of the central nervous system (CNS) (32, 33), thereby resetting thermoregulatory control. By utilizing local anesthetics to block the cervical sympathetic ganglia, SGB not only directly inhibits peripheral sympathetic outflow but has also been demonstrated to modulate CNS nuclei integral to thermoregulation–including the hypothalamus, the amygdala, and the insular cortex–via multisynaptic pathways. This centrally mediated effect reduces nerve growth factor (NGF) levels and attenuates cerebral NE release, consequently restoring core body temperature stability and normalizing the thermoneutral zone (32, 34, 35). Specifically, the decline in perimenopausal estrogen levels precipitates an elevation in NGF concentrations, which subsequently induces cortical sympathetic sprouting and increases cerebral NE levels. This cascade ultimately narrows or obliterates the thermoneutral zone within the hypothalamic thermoregulatory center, such that minute fluctuations in core body temperature precipitate hot flashes (32, 36); SGB reverses this pathological cascade by reducing NGF levels (36, 37), and elevates the sweating threshold by modulating the NE-mediated depression of thermoregulatory set-points (34, 38). Neuroimaging evidence further suggests that SGB modulates cerebral hemodynamics, characterized by extracranial vasodilation and increased blood flow; this may exert an adjunctive effect by regulating perfusion within thermoregulatory regions (39, 40). Anatomically, second- and third-order synaptic connections exist between the stellate ganglion and the hypothalamic neuroendocrine network; SGB may therefore indirectly modulate the function of luteinizing hormone-releasing hormone (LHRH)-secreting cells via regulation of these pathways (39). In terms of clinical efficacy, a randomized, sham-controlled trial by Walega et al. demonstrated that ultrasound-guided injection of 5 mL of 0.5% bupivacaine at the C6 level reduced the frequency of moderate-to-severe hot flashes in postmenopausal women by 52% over 6 months (relative risk 0.50; 95% CI 0.35–0.71; *P* < 0.001); concomitant objective assessment via skin conductance monitoring corroborated a significant 29% reduction (40). In a cohort of breast cancer survivors, a 24-weeks follow-up study by Haest et al. involving 34 patients revealed that a single injection of 10 mL of 0.25% levobupivacaine resulted in a 64% reduction in hot flash scores within the first week (95% CI 49%–74%), with a sustained reduction of 47% at 24 weeks (34). A series of studies by the Lipov group further elucidated the cumulative nature of the therapeutic efficacy; their case series (*n* = 6) demonstrated that the remission period following the initial block ranged from 2 to 5 weeks, whereas repeated blocks extended this interval to 4–18 weeks, reaching a maximum of 48 weeks (38), Additionally, a prospective follow-up of 13 breast cancer survivors (mean duration: 42.6 weeks) confirmed a significant reduction in the weekly frequency of hot flashes from a baseline of 79.4–6.9 episodes (*P* < 0.0001) (33). Notably, a head-to-head randomized controlled trial (*n* = 40) indicated that SGB demonstrated efficacy comparable to paroxetine in improving hot flash scores and sleep quality indices, while circumventing the risks associated with CYP2D6 enzyme inhibition and systemic drug exposure (41). In terms of safety, SGB is generally well-tolerated, with an incidence of severe complications of only 1.7 per 1,000; common adverse events include transient Horner syndrome, hoarseness, and dizziness (Table 3). Future research should focus on optimizing blockade protocols and injection intervals, conducting large-scale, long-term, blinded controlled trials to definitively establish its clinical role, and further exploring its therapeutic value in special populations, particularly breast cancer survivors.

**TABLE 3:** Comparison of the efficacy and duration of different stellate ganglion block regimens for the management of hot flashes.

Study	Medication regimen	Follow-up duration	Percentage reduction in hot flash frequency (%)	Repeated block regimen
Effects of stellate ganglion block on vasomotor symptoms: findings from a randomized controlled clinical trial in postmenopausal women	0.5% bupivacaine (5 mL)	6 months	A 52% reduction was observed in the SGB group, compared with a 4% reduction in the sham group.	Not reported
Stellate ganglion block for the management of hot flashes and sleep disturbances in breast cancer survivors: an uncontrolled experimental study with 24 weeks of follow-up	0.25% levobupivacaine (10 mL)	24 weeks	The maximum reduction was observed at week 1 (64%), followed by a gradual recovery; however, levels remained 47% below baseline at week 24.	Treatment was initiated with a unilateral block, followed by contralateral rescue therapy (up to a maximum of three sessions) in the event of an inadequate response. Each procedure utilized 10 mL of 0.25% levobupivacaine, with inter-block intervals determined by the timing of symptom recurrence.
Stellate ganglion blockade provides relief from menopausal hot flashes: a case report series	5 mL of 0.375% bupivacaine	The maximum individual follow-up duration was ≥48 weeks (approximately 11 months), with the remaining patients followed for 3–18 weeks.	Absolute values or percentage reduction in the frequency of daily hot flashes	Subsequent blocks were administered when symptoms returned to a “mild” severity level.

### SGB and insomnia

Stellate ganglion block exerts significant therapeutic efficacy in the management of sleep disorders via a multi-system regulatory network; its core mechanisms involve the restoration of autonomic balance, the regulation of neuroendocrine homeostasis, and the inhibition of neuroinflammation. At the level of neuromodulation, SGB attenuates central sympathetic tone by blocking the cervical sympathetic ganglia, inhibiting NE release, and enhancing vagal activity, thereby restoring the balance between the sympathetic and parasympathetic nervous systems (42, 43). Regarding neurotransmitter and endocrine function, SGB upregulates the secretion of serotonin (5-HT), neuropeptide Y (NPY), and melatonin, while concurrently inhibiting hyperactivation of the hypothalamic-pituitary-adrenal (HPA) axis; this results in significant reductions in cortisol, epinephrine, and adrenocorticotropic hormone levels (42–45), thereby ameliorating sleep-wake cycle disturbances. In terms of anti-inflammatory and neuroprotective effects, SGB downregulates the expression of proinflammatory cytokines such as IL-6, IL-1β, and TNF-α in the hippocampus and inhibits Caspase-3-mediated neuronal apoptosis, thereby attenuating neuroinflammation and cognitive dysfunction induced by surgical trauma or sleep deprivation (42, 46, 47). Clinical evidence indicates that a single ultrasound-guided injection of 3–7 mL of 0.25%–0.5% ropivacaine–with the onset of Horner syndrome serving as the marker of successful blockade–significantly improves sleep quality in patients with breast cancer, gastrointestinal malignancies, and those undergoing thoracoscopic surgery; this improvement is characterized by reduced Pittsburgh Sleep Quality Index (PSQI) scores, enhanced sleep efficiency, and prolonged N3 sleep stages (42, 45, 46). In a single-blind randomized controlled trial whose primary outcome was perimenopausal hot flashes, six consecutive daily SGB sessions (ultrasound-guided at C6, 5 mL of 0.5% ropivacaine, alternating sides) reduced PSQI scores from 10.80 to 2.14 at the 12-weeks follow-up, compared with 11.20–10.38 in the saline control group (48); similarly, in a randomized controlled trial of 128 patients with generalized anxiety disorder and sleep disturbance, adding four ultrasound-guided SGB sessions to cognitive behavioral therapy plus estazolam reduced PSQI scores from 17.92 to 5.74 at 1 month (vs. 17.86–8.03 in the control group receiving CBT plus estazolam alone) and improved sleep efficiency to 90.23% (43). Furthermore, alternative modalities such as non-invasive linear polarized light irradiation of the stellate ganglion (LI-SG) and pulsed radiofrequency ablation have demonstrated comparable efficacy (49, 50). Although existing randomized controlled trials are limited by small sample sizes and significant heterogeneity in drug concentration protocols, SGB, as a non-pharmacological intervention possessing both anti-inflammatory and neuromodulatory properties, offers a safe and effective clinical therapeutic pathway for patients with postoperative and chronic insomnia.

### SGB and cerebrovascular disease

Stellate ganglion block exerts pleiotropic effects in the treatment of cerebrovascular diseases by blocking the cervical sympathetic chain; its core mechanisms encompass hemodynamic improvement, neuroprotection, and the regulation of microcirculation. Specifically, SGB significantly increases blood flow velocity in the ipsilateral common carotid artery. In seven patients with head and neck pain, magnetic resonance bolus-tracking showed that ipsilateral common carotid artery flow velocity increased from 23.6 ± 6.45 to 40.4 ± 4.83 cm/s (*P* < 0.002) 15–30 min after the administration of 6–8 mL of 1% mepivacaine, whereas vertebral artery velocity remained unchanged, suggesting that the increase derives predominantly from extracranial vasodilation (51). Separately, Doppler studies in patients with sudden deafness demonstrated increased ipsilateral carotid and vertebral artery blood flow after SGB, with an age-dependent attenuation of the response (52). At the molecular level, SGB downregulates plasma endothelin-1 (reducing levels from 53.68 ± 6.68 to 36.70 ± 4.85 pg/mL) and upregulates calcitonin gene-related peptide (increasing levels from 633.32 ± 55.61 to 746.16 ± 84.53 pg/mL); concurrently, by inhibiting Bax and enhancing Bcl-2 protein expression, it exerts dual vasodilatory and neuroprotective effects in models of subarachnoid hemorrhage (53). Hemodynamic assessment indicates that SGB elevates cerebral perfusion pressure while reducing vascular resistance; in 20 patients with brachial plexus injury receiving 10 mL of 2% lidocaine, estimated cerebral perfusion pressure increased from 59 (51, 67) mmHg to 70 (60, 78) mmHg, without compromising cerebral autoregulation (54). Notably, SGB exerts differential regulatory effects on extracranial and intracranial vessels. In 19 healthy volunteers administered 6 mL of 1% mepivacaine, the diameter of the ipsilateral external carotid artery increased significantly by 26.5% (*P* < 0.001), whereas intracranial vessels, with the exception of the ophthalmic artery, remained largely unaffected (55). A randomized controlled trial by Zeng et al. found that in 84 patients with cerebral small vessel disease, a 20-days course of daily SGB (landmark-based paratracheal approach; 1.5 mL of 2% lidocaine plus vitamin B12) adjunctive to standard rehabilitation significantly improved swallowing function (penetration–aspiration scale) and cognitive function (MMSE) compared with rehabilitation alone (56). In the management of cerebral vasospasm following subarachnoid hemorrhage, 10 mL of 0.5% bupivacaine significantly reduced peak systolic velocity in the middle cerebral artery (from 150.25 to 140.30 cm/s, *P* = 0.005) and dilated the M1 segment; however, the rate of clinical neurological improvement was limited to 25%, a discrepancy attributed to clinical-radiological dissociation resulting from the lack of significant improvement in microvascular perfusion (57). Furthermore, SGB exerts a significant impact on retinal microcirculation: in 11 healthy subjects, blood flow to the optic nerve head increased by 106.8% (*P* = 0.001) 30 min after the administration of 6–8 mL of 1% mepivacaine (58), and it has been shown to augment blood flow in grafted vessels following vascular reconstruction (59). Early mid-20th-century reports suggested neurological improvement when SGB was administered within hours of cerebral embolism (60). However, these observations predate modern trial methodology and are presented as historical context only.

### SGB and psychiatric disorders

Post-Traumatic Stress Disorder is a devastating and prevalent psychological disorder characterized by excessive fear memories resulting from severe trauma. SGB intervenes in the consolidation process of fear memories in PTSD by inhibiting the neural circuit from the locus coeruleus-noradrenergic (LC-NE) system to the basolateral amygdala (BLA), thereby reducing central NE release and sympathetic nervous system hyperactivity (61). Animal studies utilizing pseudorabies virus-mediated retrograde tracing, fiber photometry, and electrophysiological recording have confirmed that SGB significantly reduces the excitability of LC-NE neurons and BLA glutamatergic neurons, thereby specifically impairing memory consolidation without affecting retrieval when administered within 30 min following fear memory training (61). Clinical studies have further validated this mechanism; a secondary analysis of the randomized controlled trial involving 113 active-duty service members found the largest treatment effect in the PTSD symptom cluster of “alterations in arousal and reactivity” (hypervigilance, difficulty concentrating, and sleep disturbance), with the highest odds ratio for cluster remission among all CAPS-5 clusters (OR = 4.58); however, this was an exploratory, hypothesis-generating analysis and the 95% confidence interval (0.96–21.90) crossed unity (62). This finding is consistent with the conclusions of a survey of behavioral health clinicians, in which 96% of respondents considered SGB to be most effective for symptoms related to arousal and reactivity (63). Systematic reviews indicate that 88% of published studies have applied SGB to the treatment of PTSD. Clinically, an ultrasound-guided right-sided approach is commonly utilized, involving the injection of 5–7 mL of 0.5% ropivacaine at the C6 vertebral level; ipsilateral Horner syndrome serves as the marker of successful blockade, and the administration of 1–2 blocks is recommended (64, 65). In terms of efficacy, uncontrolled case series indicate that over 70% of patients achieve a clinically meaningful improvement–defined as a ≥10-point reduction on the PTSD Checklist–Military version (PCL-M)–with response rates of 78.6% at 1 week, 81.7% at 1–2 months, and 73.5% at 3–6 months in the largest series (*n* = 166) (65), whereas a meta-analysis confirmed that SGB results in a mean reduction of 6.24 points in Clinician-Administered PTSD Scale (CAPS) scores (95% CI: −10.71 to −1.78) (66). However, it is important to note that these findings are primarily derived from observational studies and case series with inherent selection bias, highlighting the need for adequately powered, rigorously designed RCTs to establish definitive efficacy. Notably, in a retrospective analysis of 205 patients, 20 did not respond to right-sided SGB; among the 10 who subsequently received left-sided SGB, 90% responded favorably (mean PCL-5 improvement 28.3 points), indicating that at least 4.4% of the overall cohort may benefit from switching laterality (67). Bilateral blocks should be avoided on the same day to preclude the risks of bilateral recurrent laryngeal nerve block and bilateral phrenic nerve involvement (see Section “Safety profile and complications”). In terms of safety, common adverse effects include transient hoarseness, dysphagia, and Horner syndrome; in a questionnaire survey of approximately 45,000 blocks, severe complications occurred at a rate of 1.7 per 1,000, and a systematic review of 260 reported adverse events confirmed that serious events–such as seizure following vertebral artery injection, hematoma requiring airway intervention, and pneumothorax–are rare, indicating that the procedure is generally well-tolerated (68, 69). However, significant limitations remain in the current evidence base: first, conclusions from RCTs are mixed: the first, small RCT (*n* = 42) found no benefit over sham (70), whereas the subsequent multicenter RCT in 113 active-duty service members–funded by the U.S. Department of Defense–demonstrated a statistically significant CAPS-5 reduction with two SGB treatments 2 weeks apart (adjusted mean change −12.6 vs. −6.1 points, *P* = 0.01) (71). A 2017 VA evidence brief had rated the pre-2020 evidence base as “insufficient” to estimate the effect (72), most studies remain constrained by limited sample sizes, and long-term efficacy is unclear. Second, prospective data supporting the optimal administration frequency, duration of effect, and individualized selection of laterality are lacking.

Stellate ganglion block provides an effective intervention for anxiety and depressive disorders via multiple mechanisms, including the restoration of autonomic balance, inhibition of hypothalamic-pituitary-adrenal (HPA) axis hyperactivation, regulation of central monoamine neurotransmitters, and anti-inflammatory and antioxidant effects. Specifically, SGB blocks hyperactivity of the cervical sympathetic chain, reduces intracranial vasospasm, and improves cerebral perfusion, while simultaneously decreasing the levels of stress hormones such as epinephrine, NE, and cortisol. At the central level, SGB modulates mood by reducing NE release and increasing concentrations of serotonin (5-HT) and neuropeptide Y; furthermore, it inhibits the hypoxia-inducible factor-1α (HIF-1α)/NLRP3 inflammasome signaling pathway, thereby attenuating neuroinflammation induced by microglial activation (43, 73–75). In terms of clinical application, a retrospective study on postpartum depression (*n* = 98) demonstrated that the ropivacaine SGB group achieved superior improvements in HAMD, EPDS, and PSQI scores compared to the escitalopram group, accompanied by lower stress hormone levels (76); for treatment-resistant depression, the LIFT-MOOD pilot randomized double-blind trial (*n* = 10) preliminarily validated the feasibility of SGB, although it failed to establish definitive efficacy due to sample size limitations (77); animal experiments further revealed that repeated SGB reversed the activation of the HIF-1α/NLRP3 pathway following thalamic stroke in rats, thereby preventing and treating anxiety- and depression-like behaviors (74, 75).

### SGB and COVID-19

The therapeutic role of SGB in the treatment of post-COVID-19 sequelae is primarily realized through resetting the autonomic nervous system, improving cerebral blood perfusion, and modulating neuroimmune inflammation. Its mechanisms of action encompass: (1) blocking hyperactivation of the cervical sympathetic chain, reducing levels of proinflammatory cytokines (TNF-α, IL-6) and enhancing IL-10 expression, thereby breaking the “sympathetic hyperactivity-inflammation-symptom” vicious cycle (78, 79); (2) increasing blood supply to the cerebral cortex and regions associated with olfaction and gustation, facilitating the recovery of cranial nerve function (80, 81); (3) regulating neuroimmune feedback loops via vagus nerve-mediated central effects, thereby ameliorating cognitive dysfunction and fatigue symptoms (82, 83). Clinical evidence is primarily derived from case reports and retrospective cohort studies. Andrassy et al. treated two patients with Long COVID of over 18 months’ duration using ultrasound-guided unilateral SGB (0.5% bupivacaine combined with betamethasone); evaluations using the Visual Analog Scale (VAS) and Brief Pain Inventory (BPI) demonstrated sustained improvements in cognitive function and fatigue, although headaches recurred due to the chronic nature of the condition (82). Alnatour et al. performed fluoroscopy-guided bilateral SGB (using a mixture of 0.25% bupivacaine and dexamethasone) on two patients, resulting in near-complete remission of the 10 most common Long COVID symptoms (including fatigue, dyspnea, myalgia, and olfactory dysfunction) within a 3-months follow-up period (83). Retrospective cohort studies further validate its efficacy: a study involving 52 patients showed that 55.8% reported at least short-term symptom improvement, with brain fog, fatigue, dizziness, and headache exhibiting the most significant improvements (median score reduction of 3–4.5 points), although the duration of improvement was highly heterogeneous (84); another retrospective study of 41 patients reported that 86% of patients experienced improvement in at least one symptom, with remission rates for fatigue and brain fog reaching 77% and 79%, respectively (79). It is important to note that long-term efficacy remains unclear, the duration of symptom improvement is highly heterogeneous, and 25% of patients may experience adverse reactions such as exacerbation of headache or fatigue (84).

### SGB and sympathetically mediated peripheral disorders

Beyond the domains discussed above, SGB has a long history in sympathetically mediated peripheral disorders. In hyperhidrosis–particularly craniofacial hyperhidrosis–repeated SGB can produce rapid but short-lived anhidrosis lasting hours to weeks; a small series found only transient benefit for palmar hyperhidrosis with local anesthetic alone, and more durable responses have required botulinum toxin injection into the stellate ganglion or thoracoscopic sympathetic block, so SGB is best regarded as a temporizing or prognostic measure in this setting (85, 86). In Raynaud’s phenomenon, a prospective series of 40 patients (200 blocks) showed a 62.7% rise in the perfusion index within 5 min of SGB, sustained above baseline at 2 weeks, accompanied by significant pain reduction (87); caution is warranted in systemic sclerosis, where a contralateral “steal” reduction in digital blood flow has been reported (88). Finally, SGB is a classic rescue intervention for acute upper-limb ischemia, including ischemia caused by accidental intra-arterial injection of drugs intended for intravenous administration (historically thiopental): by abolishing sympathetic vasoconstriction, SGB relieves arterial spasm and opens collateral flow. This strategy has been recommended–alongside intra-arterial vasodilators and anticoagulation–since the foundational report of Stone and Donnelly, although contemporary reviews note that outcome evidence for sympathetic blockade remains limited and must be weighed against the risks of concurrent anticoagulation (89–92).

### Safety profile and complications

The most feared acute complication of SGB is accidental intravascular injection, particularly into the vertebral artery. Because the vertebral arterial minimum convulsant dose of lidocaine is only approximately 4% of the intravenous toxic dose (93), even 1–2 mL of local anesthetic can cause immediate loss of consciousness, convulsions, respiratory arrest, or cardiac arrhythmia; transient locked-in syndrome from vertebrobasilar toxicity has also been reported (69). Importantly, negative aspiration does not exclude intravascular needle placement (94), and seizures and even cardiac arrest have occurred despite ultrasound guidance (95). In a questionnaire survey of approximately 45,000 blocks, severe complications occurred at a rate of 1.7 per 1,000, most being central events (convulsions) (68); prevention relies on real-time imaging with color Doppler, test dosing, incremental injection, and full resuscitation preparedness consistent with the ASRA practice advisory on local anesthetic systemic toxicity (96). Phrenic nerve block with ipsilateral hemidiaphragmatic paralysis can occur when injectate spreads along the prevertebral fascia to the phrenic nerve on the anterior scalene surface. It was reported in approximately 2% of patients receiving large-volume landmark-based blocks and in one case among 260 adverse events in a systematic review (69); the true incidence under low-volume ultrasound guidance is unknown because systematic diaphragmatic assessment has rarely been performed. Unilateral paralysis is usually asymptomatic in healthy individuals but can reduce pulmonary function by approximately 25% and may be hazardous in patients with respiratory compromise or contralateral diaphragmatic dysfunction. Together with the risk of bilateral recurrent laryngeal nerve block, this is the principal reason that same-day bilateral SGB should be avoided (97).

Ultrasound guidance reduces complications through several mechanisms. First, it provides real-time visualization of vulnerable structures: the carotid and vertebral arteries (including variants in which the vertebral artery lies outside the C6 foramen, found in a clinically relevant minority of subjects) (98), the “serpentine” inferior thyroid artery implicated in retropharyngeal hematoma (99), the esophagus (whose variable position has led to esophageal puncture under landmark techniques) (99), the thyroid, and the pleural dome (100). Second, it permits a substantial reduction in injectate volume: cadaveric CT work shows that 5 mL achieves ideal C4–T3 spread whereas 20 mL disperses unpredictably toward the phrenic and recurrent laryngeal nerves (101), and randomized dose-comparison data confirm that 4 mL is as effective as 8 mL under ultrasound guidance (102) –smaller volumes plausibly reduce phrenic and laryngeal involvement. Third, large-scale peripheral nerve block data associate ultrasound guidance with an approximately four-fold reduction in local anesthetic systemic toxicity (OR 0.23) (103). Nevertheless, ultrasound does not eliminate risk–vertebral artery injection with seizure or cardiac arrest has occurred under ultrasound guidance (95) –so Doppler screening, test dosing, and incremental injection remain mandatory.

## Conclusion and future perspectives

**FIGURE 1:**
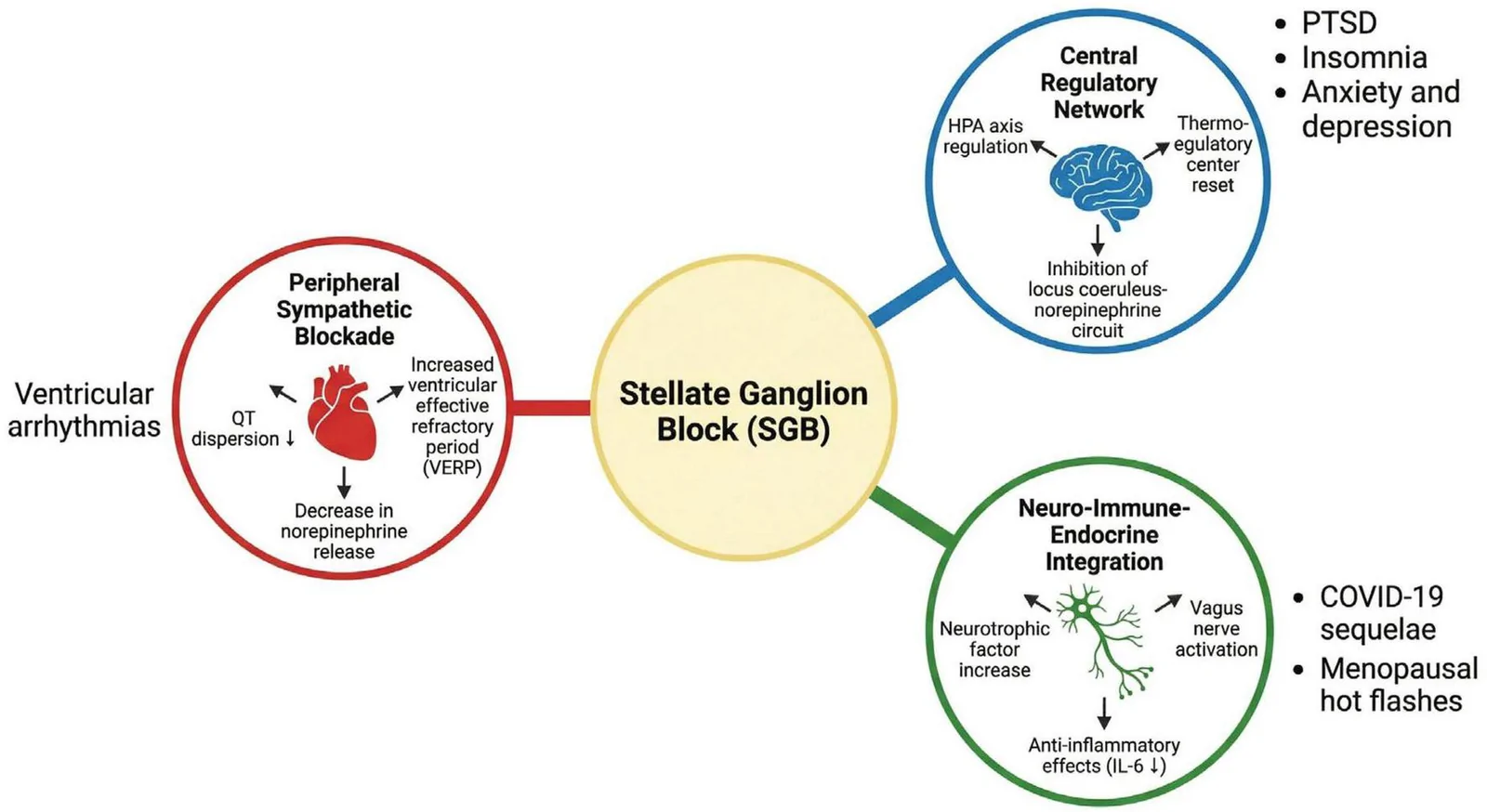
This schematic diagram systematically illustrates the multidimensional mechanisms and clinical applications of stellate ganglion block (SGB): peripheral sympathetic block (red circle): this pathway focuses on the treatment of ventricular arrhythmias by prolonging the ventricular effective refractory period (VERP), reducing norepinephrine (NE) release, and decreasing QT dispersion. Central regulatory network (blue circle): this mechanism involves HPA axis modulation, inhibition of the locus coeruleus-norepinephrine (LC-NE) pathway, and resetting of the thermoregulatory center. It is indicated for PTSD, insomnia, anxiety, and depression. Neuro-immuno-endocrine integration (green circle): this pathway targets long COVID (post-COVID-19 sequelae) and menopausal hot flashes by increasing neurotrophic factors, activating the vagus nerve, and exerting anti-inflammatory effects (e.g., reducing IL-6 levels).

Stellate ganglion block has expanded from the traditional realm of pain management to encompass a variety of non-pain disorders, emerging as a pivotal intervention for the modulation of autonomic dysfunction. Current evidence indicates that SGB demonstrates significant adjunctive therapeutic value in conditions such as arrhythmias, perimenopausal hot flashes, insomnia, cerebrovascular diseases, psychiatric disorders, and post-COVID-19 sequelae (Figure 1). This is achieved through multidimensional mechanisms, including blocking the cervicothoracic sympathetic chain, inhibiting excessive catecholamine release, restoring autonomic balance, regulating neuroendocrine homeostasis, and suppressing neuroinflammatory responses. The widespread adoption of ultrasound-guided techniques has greatly enhanced procedural safety and precision, reducing the incidence of severe complications to below 0.5% and paving the way for broad clinical application. However, with the exception of small-sample RCTs in areas such as hot flashes, PTSD, and insomnia, the application of SGB in other conditions–including arrhythmias, cerebrovascular diseases, and post-COVID-19 sequelae–relies primarily on cohort studies or case reports. Furthermore, existing RCTs are limited by insufficient sample sizes and substantial protocol heterogeneity, resulting in significant variability in efficacy and a lack of clarity regarding optimal intervention strategies. Consequently, there is an urgent need for more rigorously designed, multicenter collaborative RCTs to establish the definitive efficacy and safety of SGB across various indications. Simultaneously, the mechanisms underlying the medium- to long-term effects of SGB–mediated by central circuit modulation (e.g., the locus coeruleus-amygdala pathway and the hypothalamic thermoregulatory center) and neuroplasticity–remain unelucidated. Future research must integrate optogenetics, molecular imaging, and multi-omics technologies to deeply characterize the mechanisms of peripheral-central crosstalk, thereby laying the theoretical foundation for the transition of SGB from empirical treatment to mechanism-based precision intervention.
